# Defect Classification Using Postpeak Value for Pulsed Eddy-Current Technique

**DOI:** 10.3390/s20123390

**Published:** 2020-06-16

**Authors:** Jiuhao Ge, Chenkai Yang, Ping Wang, Yongsheng Shi

**Affiliations:** 1 Nondestructive Detection and Monitoring Technology for High Speed Transportation Facilities, Key Laboratory of Ministry of Industry and Information Technology, Nanjing University of Aeronautics and Astronautics, Nanjing 210016, China; yangchenkai@nuaa.edu.cn (C.Y.); zeit@263.net (P.W.); sys1048@126.com (Y.S.); 2 JSPS International Research fellow, Graduate School of Engineering, Tohoku University, 6-6-01-2, Aramaki Aza Aoba, Aoba-Ku, Sendai 980-8579, Japan; 3 Railway Infrastructure Inspection Center, Chinese State Railway Group Co., Ltd., Beijing 100081, China

**Keywords:** nondestructive testing, time domain, absolute signal, TMR sensor

## Abstract

In this paper, a feature termed as the postpeak value is proposed for the pulsed eddy current technique (PECT). Moreover, a method using the postpeak value is proposed to classify surface and reverse defects. A PECT system is built for verification purposes. Experiment results prove that the postpeak feature value has better performance than that of the traditional peak value in the case of reverse defect detection. In contrast, the peak value is better than the postpeak value in the case of surface defect detection. Experiment results also validate that the proposed classification algorithm has advantages: classification can be achieved in real time, the calculation process and results are easy to understand, and supervised training is unnecessary.

## 1. Introduction

The eddy current testing method has long been used for nondestructive testing. However, traditional single-frequency eddy current testing cannot distinguish between surface and nonsurface defects [1,2]. To achieve the simultaneous detection and classification of surface and nonsurface defects, the pulsed eddy current technique (PECT) is used because of its rich frequency components [3,4]. Signal processing is a very popular PECT research topic. In most studies, a signal taken from a defect free sample is used as a reference signal. Then, a differential signal is obtained by subtracting the reference signal from the tested one. In addition, the time-, frequency-, and time–frequency-domain methods are used to analyze the differential signal of PECT and the amounts of extracted signal features.

Time-domain features, such as peak value, time to peak, rising time, and zero crossing time, are widely used [5,6]. Yang et al. [7], and Angani et al. [8] investigated the detection of fatigue cracks and thickness using the peak value. Xu et al. [9] found that the time to peak feature is independent of lift-off distance and has good robustness. Yang et al. [10] theoretically discovered the physics of the time to peak feature. The number of unknown variables that can be resolved is proportional to the number of uncorrelated features. In addition to these traditional features, studies also proposed some new features, such as lift-off intersection point, separation time, and rising point, to reduce lift-off influence and improve detection sensitivity [5,11,12,13,14].

For frequency-domain features, the amplitudes and phases of the selected frequencies obtained by Fourier transform were used to identify defects [15,16]. Zeng et al. [17] used frequency-domain features to identify the location, radius, and height of defects. Fan et al. [18] used the phases of spectral response to serve as robust features for thickness evaluation. In addition to the time- and frequency-domain-analysis methods, some time–frequency decomposition tools, such as wavelet, empirical-mode decomposition, and Rihaczek distribution, were also used quite often [19,20,21,22].

An important application of PECT is classifying surface and nonsurface defects [23]. Peak value and time to peak features are usually used as the inputs of the classification method. Basically, there are two major groups of classification methods, supervised and unsupervised. For supervised methods, principal component analysis, artificial neural networks, and support vector machines are used. However, these methods require known data and model training [24]. For unsupervised methods, K-means and Bayes methods are used, but the calculation processes are complex for users to understand. So, a user-friendly and easy real-time defect-classification method is needed. At the same time, the peak value and time to peak features are not sufficient for infield inspection. More information is often required for quantification purposes [19].

All of the above studies mainly used the differential signal. However, an eddy current induced by PECT diffuses into the conductive metal with a time delay [25], which means that the absolute signal of PECT, especially a magnetic signal, can be used to “see through” conductive overburden. Thus, more researches considering the absolute signal are needed.

The main aim of this paper was to propose a new feature of absolute signal for PECT. The new feature, called the postpeak value, was more sensitive than the peak value when detecting reverse defects. In contrast, the peak value was better than the postpeak value in the case of surface defects detection. Using this phenomenon, a brief algorithm was developed to classify surface and reverse defects. The advantages of this algorithm were that classification can be achieved in real time, the calculation process and results were easy to understand, and supervised training was unnecessary.

## 2. Postpeak Value for PECT

In this section, the principle of the postpeak value is introduced on the basis of theoretical equations. Then, the simulation method is used to interpret the method for determining postpeak value in the response of PECT, and the advantage of the postpeak value over the peak value.

### 2.1. Principle of Postpeak Value

The electromagnetic field enters a conductor in a dynamical process. The expression for the electromagnetic wave equation in terms of magnetic field *B* within a material is

(1) $$\nabla^{2}B=\mu \varepsilon \frac{\partial^{2}B}{\partial t^{2}}+\mu \sigma \frac{\partial B}{\partial t},$$

where σ is conductivity, μ is permeability, and ε is permittivity.

For a good conductor, where ωε≪σ, ω is the excitation frequency. Thus, the first term can be neglected. With the assumption of isotropic conductivity and permeability, Equation (1) can be simplified as

(2) $$\frac{\partial B}{\partial t}=\frac{1}{\mu \sigma }\nabla^{2}B.$$

Rough estimates can be made as

(3) $$\frac{\partial B}{\partial t}\sim \frac{B}{\tau_{D}}\;\mathrm{and}\;\nabla^{2}B\sim \frac{B}{l^{2}},$$

where $\tau_{D}$ is the characteristic diffusion time, and *l* is the characteristic length for the system.

The general solution to differential Equation (2) is

(4) $$B=f\left(e^{-\frac{t}{\tau_{D}}}\right),$$

In order to satisfy the estimated solutions to Equations (2) and (3), characteristic diffusion can be expressed as

(5) $$\tau_{D}\sim \mu \sigma l^{2},$$

In metal, the relationship between magnetic field *B* and current density *J* can be expressed as

(6) ∇×B=μJ.

Thus, the diffusion of the induced current density in metal is

(7) $$J=\frac{\nabla \times f\left(e^{-\frac{t}{\tau_{D}}}\right)}{\mu }.$$

Penetration depth *z* of a pulsed electromagnetic wave at given time *t* in a conducting plate is governed as [15]

(8) $$z=\sqrt{\frac{t}{\pi \mu_{0}\sigma }}.$$

From the above theoretical equations, it can be seen that the induced current diffuses into conductor with time. The response signal measured during diffusion consists of three components: signal generated by the (1) induced eddy current, (2) coil, and (3) perturbed current around the defect. During this process, all of the above components attenuate with time. The idea is that a value exists in the absolute signal that has the best detection sensitivity for nonsurface defects. In this paper, it is found that this value existed after the traditional feature peak value, called the postpeak value.

### 2.2. Determination of Postpeak Value

The steps to determine the postpeak value are described as follows:

Step 1: A calibration plate with the same material and thickness to the plate under test is fabricated. The deepest reverse notch is fabricated on the basis of the requirement (the position of the postpeak value delays with the increase of the remaining ligament. In a practical application, to improve the detectability of deep reverse defect, a compromise is needed. Normally, a requested deepest defect is announced according to the standard. Thus, the position of the postpeak value of the deepest reverse defect is fixed in detection).
Step 2: PECT probe is placed on the position without defect measuring reference signal $R_{t}^{0}$.
Step 3: PECT probe is placed on the position of defect measuring defect signal $R_{t}$. Detection sensitivity *S* of the response signal changing with time is calculated as Equation (9). Then, the biggest value in the curve is the postpeak value:
  (9) $$S=\frac{\left|R_{t}-R_{t}^{0}\right|}{R_{t}^{0}}\times 100\%.$$

**Figure 1:**
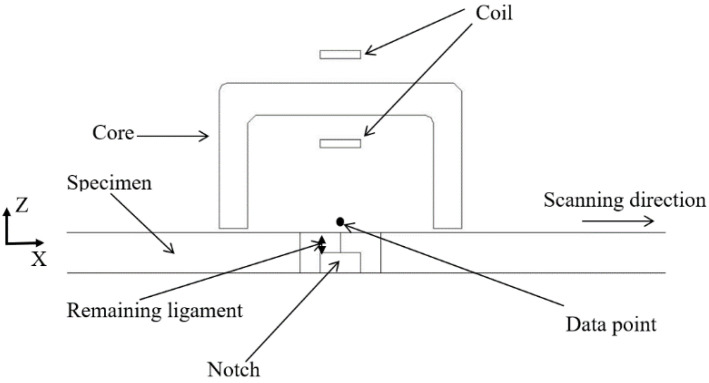
Finite element model of pulsed eddy current technique (PECT).

**Figure 2:**
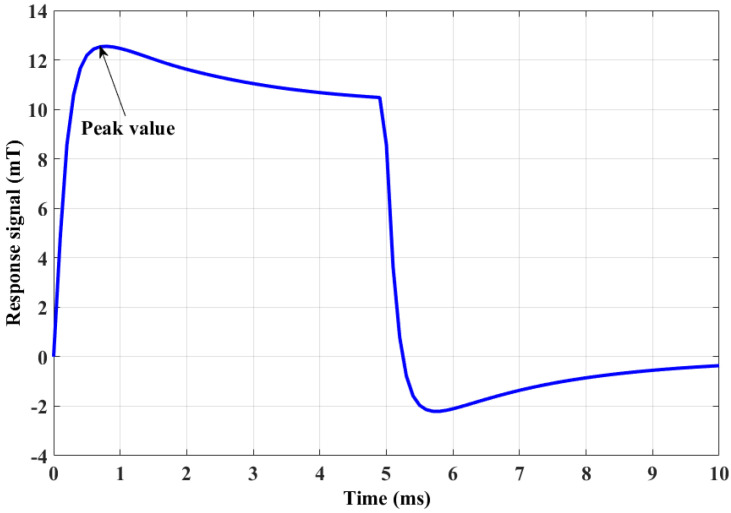
PECT response signal.

A simulation method was used to show the determination process of the postpeak value. A 3D model of PECT, consisting of an aluminum plate, coil, Mn–Zn core, air and data point was built using finite element software COMSOL 5.2a, as shown in Figure 1. The time-domain study was employed in simulation. Core parameters are shown in Table 1. The thickness of the plate was 10 mm. The turn of the excitation coil was 500, which was excited by a pulse signal with a frequency of 100 Hz, a duty cycle of 50%, and an amplitude of 8 V. The PECT probe was mounted above the plate at a lift-off distance of 1 mm. The tangential magnetic signal (X component) measured in the data point position is shown in Figure 2.

**Figure 3:**
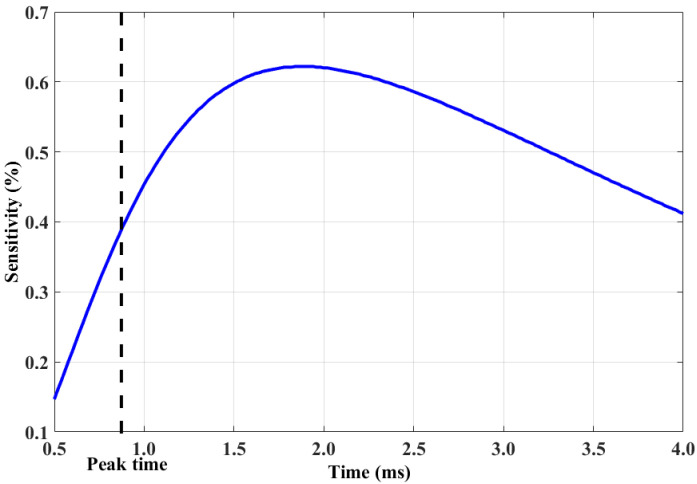
Sensitivity changes.

A reverse notch of 30 mm length, 0.5 mm width, and 5 mm remaining ligament was placed in the plate. The detection sensitivity of the response signal is shown in Figure 3, which shows that, after peak time, sensitivity initially increased and then decreased. This can be explained by the fact that the eddy current is first induced in the surface, and then diffuses into the plate. Thus, the induced current first meets the surface defect, and then the reverse defect. However, the current decays with diffusion. So, after peak time, a section exists where the measured value has a better performance for the reverse defect than that of the peak value.

**Figure 4:**
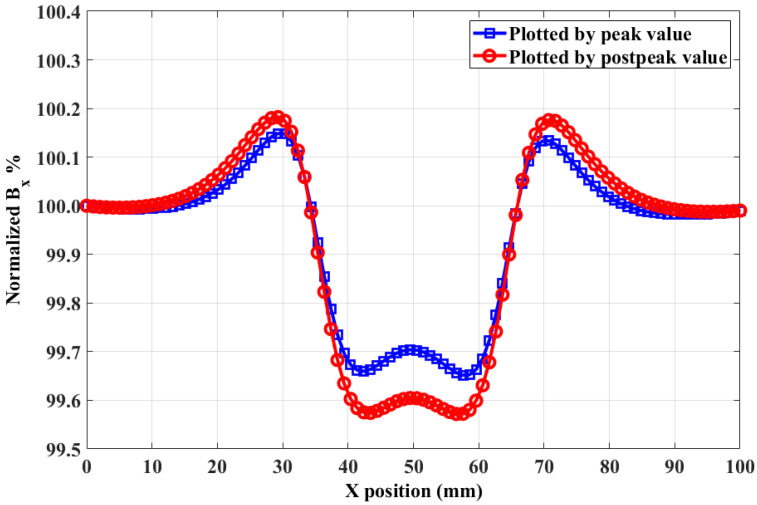
Normalized *B_x_* curves plotted by peak and postpeak values.

Then, the probe was moved along the notch with a step of 1 mm. In each position, the peak and postpeak values were recorded. The relationship between value and X position is plotted as the *B_x_* curve in Figure 4. There is a dip in the *B_x_* curve indicating the position of the notch. Moreover, the variation in the *B_x_* curve plotted by the postpeak value is bigger than that plotted by the peak value. This indicates that the postpeak value has better performance than that of the peak value in the case of reverse notch detection.

## 3. Experiment Verification

In this section, the simulation conclusion and the performance of the proposed feature are experimentally validated.

### 3.1. Experiment Device

**Figure 5:**
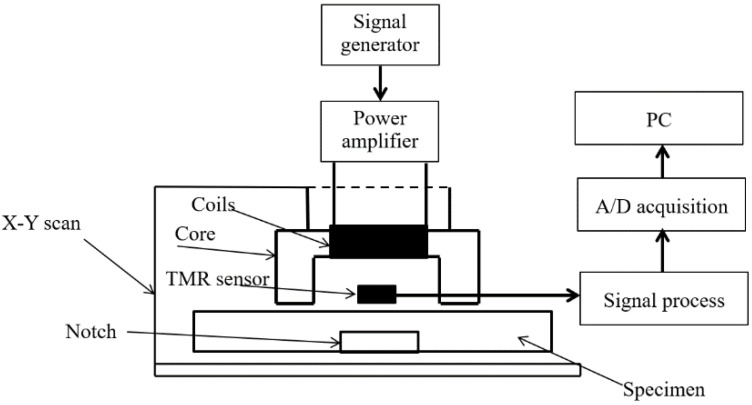
PECT system.

Figure 5 shows the PECT detection system that was built for our experiment. The parameters of the probe were the same as those in the simulation, as shown in Table 1. The probe was excited by a pulse signal with a voltage of 8 V, a duty cycle of 50%, and a frequency of 100 Hz. The turn of the coil was 500. A tunnel magnetoresistance (TMR) sensor was used as a measurement sensor with a lift-off distance of 1 mm. The tangential magnetic field signal was measured. The output of the TMR sensor was filtered, amplified, and then digitized using NI Company A/D acquisition USB6351 with a sample frequency of 50 kHz. LabVIEW software was used to store the signal of each period with the help of a trigger function.

### 3.2. Results and Discussion

**Figure 6:**
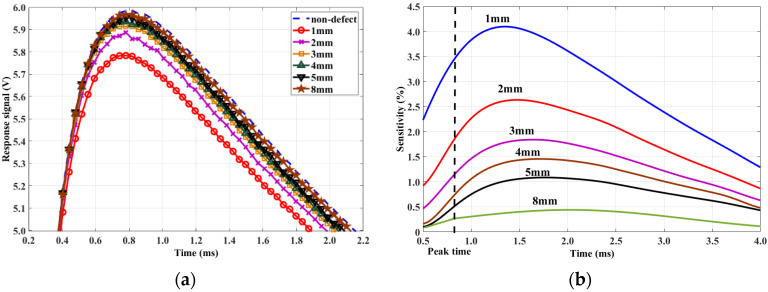
(**a**) Response signals of reverse defects and (**b**) signal sensitivity.

We measured the signals of six reverse notches (length × width = 30.0 × 0.5 mm) with a remaining ligament of 1, 2, 3, 4, 5, and 8 mm using the same method as that in the simulation. Figure 6 shows the response signal and sensitivity changes. Figure 6b shows that, after peak time, sensitivity first increases and then decreases. Thus, the phenomenon in the simulation was validated.

**Figure 7:**
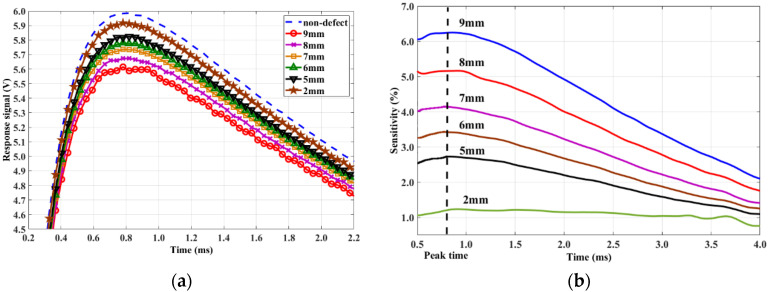
(**a**) Response signals of surface defects and (**b**) signal sensitivity.

Then, the plate was turned over, and surface notches (length × width = 30 × 0.5 mm) with a depth of 9, 8, 7, 6, 5, and 2 mm were detected. Results are shown in Figure 7, showing that the biggest sensitivity always appears at peak time.

These results reveal that, for the absolute signal, the performance of the postpeak value is better than that of the peak value when detecting reverse defects. The sensitivity of the postpeak value is always bigger than that of the peak value when detecting reverse defects. In contrast, the sensitivity of the postpeak value is always less than that of the peak value when detecting surface defects.

## 4. Defect Classification Method Using Postpeak Value

### 4.1. Classification Method Procedure

**Figure 8:**
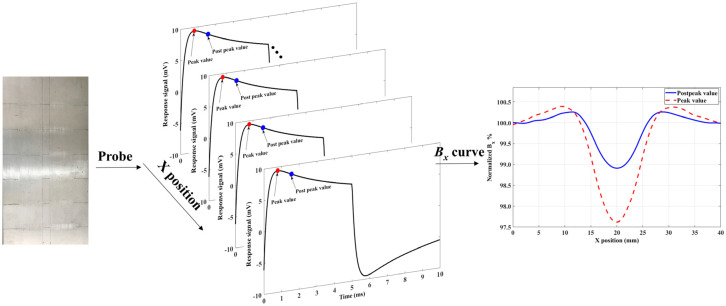
Classification method procedure.

Using the conclusion that the sensitivity of the postpeak value was always bigger than that of the peak value when detecting reverse defects, and the sensitivity of the postpeak value was always less than that of the peak value when detecting surface defects. A surface and reverse defect classification method was concluded, as shown in Figure 8. The description of the method is:

Step 1: The postpeak value for a certain detection is determined in advance according to the procedure in Section 2.2.
Step 2: When detecting defects, in each scanning point both peak and postpeak values are extracted.
Step 3: The *B_x_* curves of the measured values vs. X position are drawn.
Step 4: The difference value of the minimal values in the *B_x_* curves of the peak and postpeak values are compared. If the difference value is positive, a surface defect exists. If the difference value is negative, a reverse defect exists.

### 4.2. Classification Method Verification

**Figure 9:**
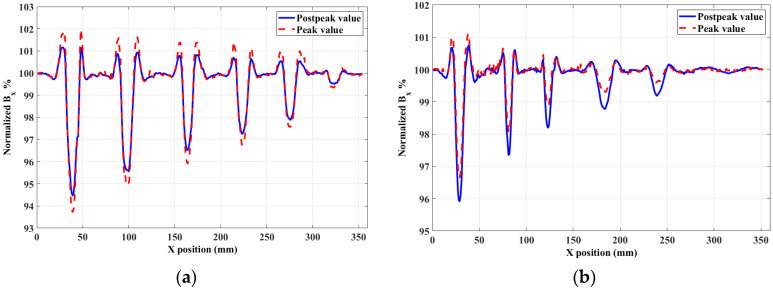
Line scanning results of (**a**) surface and (**b**) reverse defects.

To verify the proposed classification method, using the experiment device in Section 3, the probe was moved using the X–Y scan platform at a speed of 5 mm/s with a lift-off distance of 1 mm. Scan density was 10,000 data per millimeter. In each scanning point, the tangential magnetic signal was stored. The value measured at 2.2 ms was chosen as the postpeak value. The peak and postpeak values in every period were simultaneously recorded with a moving probe. Then, the *B_x_* curves of the measured values vs. X position were drawn. The normalized *B_x_* curves are shown in Figure 9.

**Figure 10:**
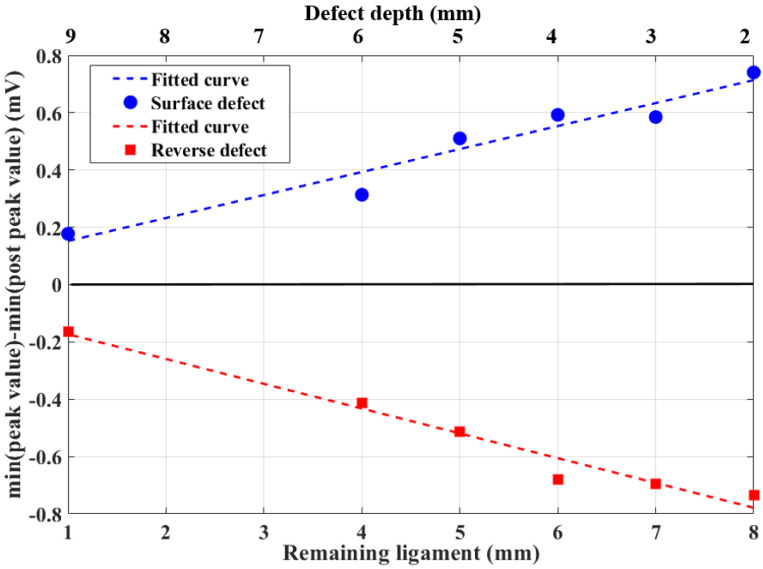
Difference value of minimum in *B_x_* curves of peak and postpeak values.

Figure 9a shows that the minimal values of the *B_x_* curve (*B*_min_) of the peak value are always lower than those of the postpeak value when detecting surface defects. Moreover, In Figure 9b, the *B*_min_ of the peak value are always higher than those of the postpeak value when detecting reverse defects. The difference values of the minimal values in the *B_x_* curves of the peak and postpeak values are shown in Figure 10. It can be directly seen that, using the difference of the minimal values in the two curves, defects could easily be classified. The advantages of this algorithm are that classification can be achieved in real time, the calculation process and results are easy to understand, and supervised training is unnecessary.

## 5. Conclusions

In this paper, we proposed a new feature called the postpeak value in the absolute signal for PECT. Experiment results indicated that the postpeak value had better detection sensitivity than the peak value when detecting reverse defects. In contrast, the peak value was more sensitive than the postpeak value in the case of surface defect detection. A brief algorithm based on this phenomenon was proposed to classify surface and reverse defects.

However, the type of probe may also have an influence on the postpeak value. Thus, the influence of the probe type will be studied in future work. The performance of the new feature for thickness measurement and lift-off influence will also be studied.

## Figures and Tables

**Table 1:** Core parameters.

Parameter	Length (mm)	Width (mm)	Height (mm)	Thickness (mm)	Conductivity (S/m)	Relative Permeability
Value	60	15	45	8	0.15	2000
